# A Psychological Exploration of the Power of Our Mindset and Its Influence on Physiological Health

**DOI:** 10.7759/cureus.52505

**Published:** 2024-01-18

**Authors:** Parmis Parsamanesh, Mykhailo Vysochyn

**Affiliations:** 1 Clinical Sciences, Saint James School of Medicine, Chicago, USA; 2 Cardiology, Saint James School of Medicine, The Valley, AIA

**Keywords:** mind-body medicine, nocebo, positive thinking, psychological and mental health, quality of life (qol), physiological health, placebo, health, influence, mindset

## Abstract

The brain is the control center for our bodies and determines our emotions, thoughts, and actions. From a psychological perspective, the mind can assist humans in manifesting a more remarkable life for themselves or hinder their abilities and result in unfulfilled potential. Considering the power of the mind, it is interesting to study the psychology of the mind and its direct influence on our physiological health. In medical terms, this effect is known as the placebo effect, where the mind and body connect on a stronger level and can assist in the betterment of an individual’s physiological health. On the contrary, the mind can also assist in deteriorating one’s physiological health by believing the medical intervention will cause them harm, known as the nocebo effect. Therefore, the mind holds much power when studying how deeply it is connected to and can influence one’s physiological health. A comprehensive literature review was conducted using the Medical Subject Headings (MeSH) terms “Mindset,” “Influence,” and “Health” on the PubMed database. The initial search generated 115 results and was narrowed by assessing each article and applying specific inclusion and exclusion criteria. As a result, nine articles were carefully selected for this review.

## Introduction and background

The placebo effect is not a panacea to an individual’s physiological health but operates on symptoms modulated by the brain. This phenomenon became part of the medical jargon in the late 18th century, and researchers continue to explore this topic to seek various opportunities to implement it in medicine [1]. For instance, it can help reduce side effects typically associated with a disease, such as nausea, dizziness, and fatigue. An individual’s mindset ultimately triggers this effect as the brain will command the body of the outcome it anticipates. Thus, the mindset has a significant effect on perception and behavior. Healthy mindset can be classified as “fixed” or “growth.” When fixed, the mind is limiting and static, whereas a growth mindset is open to more notions, encouraging individuals to challenge and improve themselves. A growth mindset is favored when examining its influence on an individual’s physiological health, supported by the studies analyzed in this systematic review. More specifically, when patients visit the physician’s office and receive a particular diagnosis, how they handle the news reflects their perception.

A Danish study in 2008 revealed that 48% of physicians had prescribed placebos a minimum of 10 times in the previous year [2]. This statistic indicates the significance and prevalence of placebos already implemented in the healthcare industry. Thus, further research on this effect and its benefits can open doors to more medical opportunities.

Stanford researchers Alia Crum and Damon J. Phillips explored the psychology of the mindset in various domains. They deduced with respect to stress that individuals who believe stress has enhancing consequences tend to be happier, healthier, and perform better during stress than those who believe it has debilitating effects [3]. This example demonstrates the power of an individual’s mindset and how it dictates their perception and ultimately reflects in their emotions, behavior, and health.

Generally, a person’s perception of one’s physiological health may result from the influence of their mentality adapted by social and environmental factors. Acknowledging the power of the mind highlights the importance of paying attention to the psychological aspect of healing and not solely the physical burden. The hypothesis explored in this review is that our mindset is mighty and can influence our physiological health positively or negatively.

## Review

Materials and methods

Data Sources and Search Strategy

**Figure 1:**
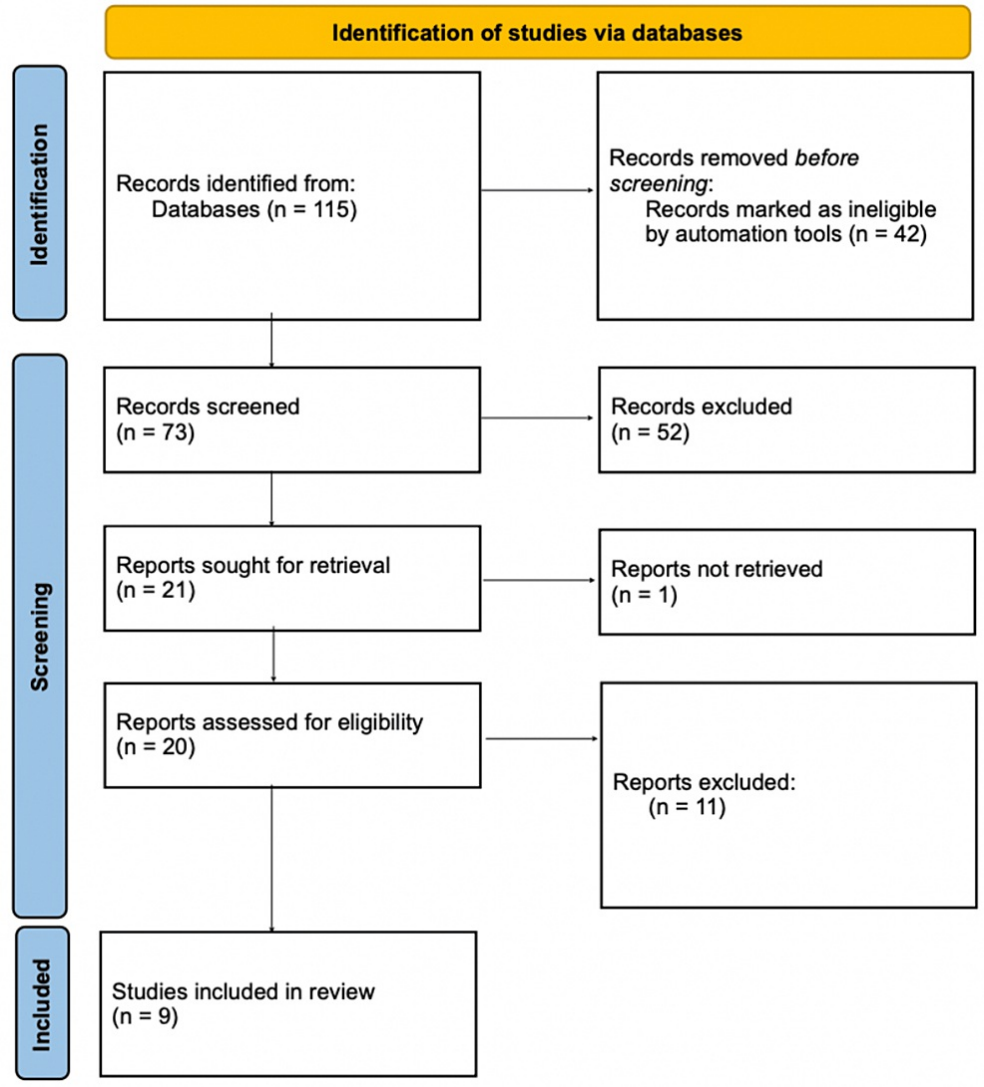
Preferred Reporting Items for systematic reviewers and meta-analysis (PRISMA) chart

A literature search was conducted using the database PubMed, and all relevant articles were identified using the Medical Subject Headings (MeSH) terms “Mindset,” “Influence,” and “Health.” The systematic review carefully followed the Preferred Reporting Items for Systematic Reviewers and Meta-analysis (PRISMA) guidelines. Specifically, this process is shown in Figure 1.

Study Selection and Eligibility Criteria

Following the stringent application of inclusion and exclusion criteria, all abstracts were intricately assessed, and the whole text of the selected articles was obtained and thoroughly analyzed. The preliminary search of the PubMed database elicited a total of 115 articles, which was narrowed down to nine when applying the necessary inclusion/exclusion criteria. To ensure the inclusion of only high-quality studies, particular inclusion and exclusion criteria were established, which were studied, analyzed, and revised multiple times. Only the articles published in the English language within the past decade focused on the positive influence of the mind on physiological health were included. Articles were excluded in cases of studies done on animals, overlapping with other articles, and unavailability of full text. The research question was established and developed, and the study was conducted only using databases with no direct patient contact. This thorough approach enabled the most pertinent databases, formulating a highly accurate, reliable, and insightful analysis.

Review

Table 1 thoroughly explains each study, and its methods, elaborates on the significance of each article, and how they are relevant to the main topic being explored.

**Table 1:** Literature review REM - Rapid eye movement, PASAT - Paced Auditory Serial Addition Test

Title of Article	Country	Design and Study Population	Findings	Conclusion
Make up your mind about food: A healthy mindset attenuates attention to high-calorie food in restrained eaters [4]	N/A	Randomized control trial n=60	The experiment focused on whether temporarily induced health versus palatability mindsets affected attention bias for food and explored whether restrained eating moderated this relation. Overweight and healthy-weight participants were either equipped with a health or palatability mindset. The associations between positive versus negative with high-calorie (palatable and unpalatable) versus low-caloric (palatable and unpalatable) food, measured in the affective priming paradigm.	A health mindset resulted in a decrease in attention bias regarding high-calorie food cues in comparison to a palatability mindset. Variables consisting of situation, internal state, and eating decision affect attention bias for food, possibly through triggering different minds, suggesting that individuals who feel ambivalent regarding high-calorie food may be more susceptible to the influence of state fluctuations on their attention processing of food cues.
Targeting Mindsets, Not Just Tumors [5]	N/A	Conceptual Research	Precise, targeted psychological interventions to shift patient mindsets can potentially transform supportive care in oncology. The psychological and social impact can be relentless with challenges not just at diagnosis but throughout treatment and recovery, extending this disease beyond its physical aspect. Psychological sciences of "wise interventions" successfully demonstrate that targeting mindsets is practical for interventions as they can easily be changed and provide notable downstream impacts.	Similar to the growth and spread of malignant cells in the body, malignant mindsets can influence the patient's experience. As physicians attempt to target these malignant cells with the most recent cutting-edge treatments, we should simultaneously attempt to deliver treatments for the psychological and social ramifications of the illness that are equally as precise.
Do health beliefs affect pain perception after pectus excavatum repair [6]?	N/A	Multi-institutional, prospective, randomized clinical trial n=50	Patients underwent a pectus excavatum repair surgery, which was minimally invasive and participated in a survey to determine whether their health mindsets were considered growth or fixed. Next, the patient's post-operative pain was followed prospectively and scored using a visual analogue scale, and the outcomes measured were according to time to oral pain medication use.	Mindset makes a difference in how patients perceive and report their pain. Interventions to ameliorate a patient's mindset can be efficacious in the future to improve pain control and patient satisfaction.
The Influence of Health Mindset on Perceptions of Illness and Behaviors Among Adolescents [7]	Suburban Midwest communities	Meta-analyses n=352 and n=124	Adolescents with a fixed health mindset are more prone to view an encounter with an illness or injury as a significant setback to health than those with a growth mindset. Specifically, healthy individuals with a fixed health mindset view an unhealthy person as less healthy, less likely to recover, and more vulnerable to additional diseases than an individual with a growth health mindset. In addition to how adolescents view another ill person, this study explored how they view themselves when facing health problems. Results showed that how they view themselves depends on their adolescent stage since they are going through a psychological development period. In the earlier stages of adolescence, the influence of caregivers, family, and peers on behaviour and decisions related to health are more pronounced. Therefore, if surrounded by people with a growth health mindset, they are more likely to encourage health maintenance and promotion behaviours. However, if surrounded by people with a fixed health mindset, they will focus less on promoting behaviour change based on their views of having a highly fixed health.	Mindset theory demonstrates that a growth mindset of health links to more adaptive attitudes toward and management of illness than a fixed mindset, which is a more fatalistic view of illness. The two studies in this research demonstrated how endorsing a fixed mindset of health can lead adolescents to view illness more negatively than holding a growth mindset. Supporting a growth mindset of health can result in better engagement in health-relevant behaviours.
Placebo sleep affects cognitive functioning [8]	N/A	n=164	Participants self-reported sleep quality, and the study informed them that the BIOPAC equipment would measure their pulse, heart rate, and brainwave frequency. However, only their brainwave frequency was measured. Some participants received news that their REM sleep was far below average (16.2%), and others were well above average (28.7%). Next, the participants completed the Paced Auditory Serial Addition Test. When informed that they had experienced below-average sleep quality the night before, the participants tended to perform worse on the PASAT despite how well they initially felt they had slept—those in the above-average sleep quality condition performed within normal limits on the PASAT. The pattern of cognitive functioning observed in the study is consistent with what one might observe if participants had experienced a poor night's sleep.	The mindset can affect cognitive states in positive and negative directions. This study demonstrated that the symptoms of sleep deprivation, including a decline in cognitive functioning, are wide-ranging. It has shown its ability to impair central processing, reduce attentional arousal, and lower cognitive functioning overall. Altering an individual's mindset or expectancy about their sleep experience could help relieve the symptoms of decreased cognitive functioning.
Effects of Mindset and Dietary Restraint on Attention Bias for Food and Food Intake [9]	N/A	n=122	Short video clips utilized served to implement a healthy or hedonic mindset. Participants performed a modified additional singleton task with pictures of high-caloric food vs. neutral pictures as unrelated distractors. The bogus taste test measured the food intake.	The experiment demonstrated that participants who scored higher on dietary restraints were more inclined to have a higher food intake. However, these effects were independent and not moderated by mindset. In addition, response latency-based attention bias for food was more prone to correspond positively with food intake in the hedonic mindset.
Aging as a Mindset: a study protocol to rejuvenate older adults with a counterclockwise psychological intervention [10]	Milan, Italy	Randomized control trial n=89	Participants from the study who embodied a more positive self-perception of ageing at baseline had better functional health throughout the study. They lived an average of 7.5 years longer than those with more negative self-perceptions of ageing. The stereotype embodiment theory describes how people age as they follow their underlying stereotypes about older people, and interestingly, individuals from cultures with predominantly strong negative beliefs about older people were more likely to experience memory problems. From a physiological point of view, the self-perception of memory decline and the stereotypic beliefs of ageing are reliable predictors of actual memory decline. It was associated with cerebral metabolic decline and brain modifications related to Alzheimer's disease, including hippocampal volume loss and accumulation of neurofibrillary tangles and amyloid plaques. Similarly, positive attitudes towards age protect against dementia, even if there is a genetic disposition.	The factors that play a role in the ageing process, including age-related stereotypes or cognitive mindset, reflect on self-perceptions and influence life and health satisfaction. This effect can either be negative or positive.
Patient Mindset and the Success of Carpal Tunnel Release [11]	N/A	n=307	Patients with carpal tunnel syndrome had their mindsets measured using the Patient Health Questionnaire-4, the Pain Casatrophizing Scale, the Brief Illness Perception Questionnaire, and the Credibility Expectancy Questionnaire. The test examined the relation between self-reported severity six months after prognosis (measured with the Boston Carpal Tunnel Questionnaire) and psychosocial aspects of mindset, adjusting for preoperative Boston Carpal Tunnel Questionnaire score, patient characteristics, and co-morbidities.	Treatment outcome comprehensibility and expectations of illness are independently associated with carpal tunnel release, demonstrating the significance of these aspects of the patient's mindset for the outcome of carpal tunnel release.
Health, pleasure, and fullness: Changing mindset affects brain responses and portion size selection in adults with overweight and obesity [12]	N/A	n=36	The study consists of three mindsets: pleasure, fullness, and health. It explored the influence of pre-meal planning by a shift in mindset in individuals who struggle to maintain average weight, i.e. are overweight or obese, to influence portion size selection and brain activity. Participants selected a portion size for a pleasure mindset that they would eat with pleasure. For the fullness mindset if they plan to be complete until dinner, and for the health mindset if they consider health aspects.	When individuals adopt a health-focused mindset (accompanied by enhanced activation of the self-control network), regardless of their weight group, they could be encouraged to reduce their portion size.

Results and discussion

This review hypothesized that our mindset's power is so strong that it influences our physiological health. After completing the extensive literature review, we accepted the hypothesis as it showed how interconnected our minds are to our bodies. The main findings include (a) providing psychological and social ramifications for a diagnosed illness will deliver positive outcomes for the patient's overall well-being [5]. (b) A growth health mindset is preferred over a fixed health mindset as the individual who is open to new ideas has a greater capacity to improve their health. In contrast, those who maintain a specific mindset before or during diagnosis and treatment are less likely to see improvement in their quality of life. Overall, one can manipulate the mind to think in ways that are beneficial or detrimental to one's health [6]. (c) Regardless of a patient's genetic disposition or current health status, having a positive mindset will, in many ways, positively influence their physiological health. So, it is essential to have a positive attitude towards any health concern to help the patient's treatment [10]. (d) There are many stereotypes surrounding various health concerns. For instance, with regards to food and dieting, if certain foods classify as healthy, then no matter the portion, an individual may feel a lack of nutrients entering the body and never reach satiety even if they have consumed the same amount of calories, they would have eaten what they may classify as unhealthy. Therefore, it is vital to acknowledge the influence such stereotypes may have when restricting food consumption [10]. Doing so allows an individual to have a healthy meal plan that suits them best. Another instance of stereotypical influence is when patients view a diagnosis such as cancer as catastrophic and heavily burdensome. Although a diagnosis may be rough in treatment or even terminal, such a mentality may provoke the patient to retreat from activities they previously engaged in, putting them in a whirlwind of negative thoughts, which may eventually lead to depression and cause even more significant problems for the patient. On the other hand, if a patient views a diagnosis as manageable or perhaps an opportunity, they may have a renewed appreciation for life and motivation to achieve personal/business goals. Thus, a patient's mindset can be a catalyst for either a negative or positive change in their life.

Another study found that macro and micro factors can alter expectancies, leading to placebo and nocebo effects. Examples of such are culture, society, and individual psychobiological traits. More specifically, a patient-centered approach rooted in demonstrating care and empathy can positively enhance a patient's experience within a clinical environment and activate psychological, social, and biological adaptations associated with the placebo phenomenon [13]. On the contrary, the nocebo effect acts utterly contrary to its opposing counterpart. Furthermore, the neurobiological basis of the placebo effect is being explored by researchers today, and two hypothesized psychological mechanisms known to mediate this effect are classical conditioning and expectancy [14]. Understanding the mechanisms of the phenomenon allows us to make more discoveries in this field that could improve patient care on many levels.

Various forms of literature, including medical publications and non-fiction novels, discuss the impact of mindset on physical health. Medical research demonstrates how science and culture align in riveting ways. The literature review of “Aging as a Mindset” highlights the stereotype embodiment theory (SET); the human mind is primarily determined by the stereotypes associated with age in this instance [10]. Many, especially the younger population, do not know this concept, as experience and studies may lead to such realization. American professor of sociology and author Morris S. Schwartz elaborates on this concept further when suffering from the terminal illness ALS in a book by Mitch Albom [15]. Going from being independent to dependent while ill without embracing its supposed cultural shame allowed him peace as he had already suffered physically. Doing so allows the patient to focus on what is in their control and not be weary of things they have no power over. Therefore, the patient can ignore the agony of mental distress caused by cultural stereotypes. If the narrative positively switches direction, the patient will not have added stress to their physical pain. Furthermore, to understand the benefits of positive thinking and improving our mental health, we must realize that positive thinking means approaching unpleasantness, in this research- a physical medical concern, more positively and productively [16].

Cultivating the skill of positive thinking while dealing with an illness can render a better quality of life with tremendous self-satisfaction and health outcomes. Positive thinking is the conscious act of finding the positive side of an act or encounter [17]. To achieve this, the patient can capitalize on the aspects of their life that bring them joy, heighten their gratitude and awareness despite all challenges, connect to others experiencing similar things, and develop wisdom or strengthen faith if applicable. Moreover, Dr. Eric Kim, who studies the phenomenon of positive thoughts and positive attitudes (also known as positive thinking) at the Harvard T.H. Chan School of Public Health, remarked, “When comparing the most optimistic to the least optimistic, the most optimistic have a reduced risk of dying from cancer, infection, stroke, heart disease, and lung disease” [17]. Thus, many doctors and researchers have highlighted the importance and power of a patient's mindset on their physiological health. Removing stress-causing factors ultimately plays a significant role as it directly affects well-being.

## Conclusions

The psychological exploration of the power of our mindsets proved influential on physiological health. As such, it can not prevent or cure a disease, however, it can influence the quality of life of the patient (in a positive or negative way depending on the patient’s mindset). Creating a positive perception of the circumstances in which the patient finds themself due to an illness is a complex and multifaceted process that begins with the first visit to the doctor. Yes, the work of a psychologist and in some cases, a psychiatrist will play a big role, but the doctor or nurse can become the initiating link. The manner in which you perceive any aspect of an illness including its symptoms, prognosis, potential risks, and care/treatment plans, will to an extent reflect on the body physiologically. Similarly, the manner in which healthcare professionals interact with and treat patients will ultimately have an influence on the perception of the patient’s health concerns.

The psychology of our mindset plays a significant role in our physiological health; thus, it is vital to address both factors when receiving care. Although implementing psychosocial interventions when dealing with various physiological health problems is wise in the standard of supportive health care, it may be difficult to manage for patients. In particular, this addition could increase the treatment timeline and be costly. One method that can facilitate the implementation of this process is providing online services, which can save patients time from constant hospital visits and allow them access to healthcare from the comfort of their homes. Furthermore, to improve patient mindsets and direct them toward a more positive and growth perspective, healthcare providers can identify those with maladaptive mindsets and help them establish more adaptive mindsets. To achieve this goal, patients and healthcare providers must work together to shift the cultural conversation around an illness, i.e., it does not overpower all aspects of one’s life; with the appropriate care instructions/treatment, patients should continue to live their lives without the negative stereotypes surrounding diseases.
